# A retrospective observational study of vCare: a virtual emergency clinical advisory and transfer service in rural and remote Australia

**DOI:** 10.1186/s12913-023-10425-7

**Published:** 2024-01-18

**Authors:** Kimberley Dean, Cynthia Chang, Erin McKenna, Shannon Nott, Amanda Hunter, Julie A. Tall, Madeline Setterfield, Bridget Addis, Emma Webster

**Affiliations:** 1 Orange Health Service, Western NSW Local Health District, 1530 Forest Road, Orange, NSW 2800 Australia; 2 Maitland Hospital, Hunter New England Local Health District, 51 Metford Rd, Metford, NSW 2323 Australia; 3 School of Rural Health, Faculty of Medicine and Health, University of Sydney, 4 Moran Drive, Dubbo, NSW 2830 Australia; 4 Western NSW Local Health District, 7 Commercial Ave, Dubbo, NSW 2830 Australia; 5 vCare Western NSW Local Health District, PO Box 739, Dubbo, NSW 2830 Australia; 6 Health Intelligence Unit, Western NSW Local Health District, Ward 22, Bloomfield Campus, Locked Bag 6008, Orange, NSW 2800 Australia

**Keywords:** Emergency care, Retrieval medicine, Rural and remote healthcare, Telehealth, Transfer medicine, Virtual medicine

## Abstract

**Background:**

Provision of critical care in rural areas is challenging due to geographic distance, smaller facilities, generalist skill mix and population characteristics. Internationally, the amalgamation telemedicine and retrieval medicine services are developing to overcome these challenges. Virtual emergency clinical advisory and transfer service (vCare) is one of these novel services based in New South Wales, Australia. We aim to describe patient encounters with vCare from call initiation at the referring site to definitive care at the accepting site.

**Methods:**

This retrospective observational study reviewed all patients using vCare in rural and remote Australia for clinical advice and/or inter-hospital transfer for higher level of care between February and March 2021. Data were extracted from electronic medical records and included remoteness of sites, presenting complaint, triage category, camera use, patient characteristics, transfer information, escalation of therapeutic intervention and outcomes. Data were summarised using cross tabulation.

**Results:**

1,678 critical care patients were supported by vCare, with children (12.5%), adults (50.6%) and older people (36.9%) evenly split between sexes. Clinicians mainly referred to vCare for trauma (15.1%), cardiac (16.1%) and gastroenterological (14.8%) presentations. A referral to vCare led to an escalation of invasive intervention, skill, and resources for patient care. vCare cameras were used in 19.8% of cases. Overall, 70.5% (*n* = 1,139) of patients required transfer. Of those, 95.1% were transferred to major regional hospitals and 11.7% required secondary transfer to higher acuity hospitals. Of high-urgency referrals, 42.6% did not receive high priority transport. Imaging most requested included CT and MRI scans (37.2%). Admissions were for physician (33.1%) and surgical care (23.3%). The survival rate was 98.6%.

**Conclusion:**

vCare was used by staff in rural and remote facilities to support decision making and care of patients in a critical condition. Issues were identified including low utilisation of equipment, heavy reliance on regional sites and high rates of secondary transfer. However, these models are addressing a key gap in the health workforce and supporting rural and remote communities to receive care.

## Background

Sparsely populated land areas dominate most countries of the globe [1]. In these areas, providing routine and specialised healthcare services is an ongoing challenge [1–7]. While new services are being implemented to address this problem, little is published which describes the patient journey from call initiation at the referring site to definitive care at the accepting site. We need to build a strong base of knowledge to understand these novel services and the role that they play in struggling communities.

In Australia, timely access to health services and specialist medical care is a challenge for rural and remote communities with patients often having to drive up to 5–6 h to access appropriate care [2–7]. Compared to metropolitan residents, people living in rural areas have poorer health outcomes, higher preventable hospitalisation rates and shorter life expectancy [3, 4]. In emergency care, where time, staffing and resources are critical to patient outcomes, mortality is up to 14% higher among rural-remote patients than among metropolitan patients [5–7].

The Modified Monash Model (MM) is commonly used in Australia to measure remoteness of location [8]. A score of MM1 refers to major cities, MM5 refers to small rural towns and MM7 refers to very remote communities. Remote areas consistently report difficulty recruiting and retaining health staff both nationally and globally [4, 9, 10]. Rural and remote areas recruit and retain insufficient numbers of general practitioners, medical specialists, dental practitioners, nurses and allied health professionals to service demand [10]. The causes of workforce shortages are complex, but relate to geographic, social, personal, structural, and educational issues. Emergency departments (EDs) in remote communities are often staffed exclusively by nurses and/or rural generalists with limited access to services such as imaging, pathology, and surgery. Accordingly, high-acuity patient presentations often exceed the local role delineation of rural hospitals and require transfer to regional hospitals (MM3) for definitive care and better health outcomes [6, 11, 12].

Services are developing across the world to address this gap, such as the virtual emergency clinical advisory support service (vCare – see below) in New South Wales (NSW) in 2006 and the medical retrieval and primary health advice model introduced in Central Australia in 2018 [13, 14]. The vCare model and evolution of the service have been previously described [13, 14]. Understanding these services which combine retrieval and telemedicine is vital [7, 13–20]. Previous studies identified that telemedicine services used in conjunction with retrieval were well received by both patients and staff members alike as they improved communication and decision making [7, 16, 17, 20]. There are few studies on the effectiveness of these services, Mathews et al. (2008) identified a decrease in retrievals and Armstrong et al. (2014) found little difference in retrieval rates in a paediatric population [15, 18]. However, there is limited data in these papers and other studies of similar services which describe the patient journey. Sri-Ganeshan et al. (2023) studied a virtual ED program in Victoria, Australia, showing excellent uptake of the service and decrease in ED admission secondary to telehealth, yet the service lacks the logistic complexity of retrieval medicine and remote sites [21]. Quantitative description of a retrieval telehealth service is the first step needed to better understand the complexities of these novel services and improve health care provided to rural and remote communities.

This study aimed to describe the vCare patient journey from call initiation at the referring site to definitive care at the accepting site. This is achieved by:

- Describing the remoteness of referring sites and accepting sites
- Describing presenting complaint and triage category
- Describing camera use in patient assessment by vCare
- Describing characteristics of patients who were referred to the service
- Describing if and how patients were transferred between sites
- Describing the escalation and de-escalation of therapeutic intervention during the patient journey
- Describing patient outcomes

## Methods

This retrospective observation study reviewed two months of consecutive vCare encounters. An encounter is defined as patient referral to vCare for clinical advice. vCare is a 24/7 virtual emergency clinical advisory service that provides specialist advice as part of the rural Clinical Emergency Response System and centralised care coordination inter-hospital transfer and logistics support across rural and remote communities in Western NSW Local Health District (WNSWLHD) [13, 14, 22]. vCare services 246,676 square kilometres (31%) of NSW and a diverse population of almost 300,000 people with an Indigenous population of 11% (compared to the state average of 3.4%) and high levels of social disadvantage [13, 22].

Healthcare services in WNSWLHD include three major regional hospitals (MM3), four procedural hospitals (MM4) and 33 smaller community hospitals or multipurpose health services (MM5-7). The 33 smaller sites are staffed by nurses located on-site and by on-call Rural Generalists who also undertake primary care duties for the local community [13, 14, 22]. Where there is no local doctor available or when the local doctor requires additional support, these hospitals are also supported by the Virtual Rural Generalist Service (VRGS) – a supplementary telehealth service that provides support for both emergency and inpatient care [23]. For clarity, transfer is defined as the relocation of a patient from the referring site to the accepting site [24] and telehealth is defined as the provision of medical services through secure telephone or video-conferencing technology while retrieval medicine is the specialty of “assessing, stabilising and transporting patients with severe injury or critical illness” [24, 25].

**Fig. 1:**
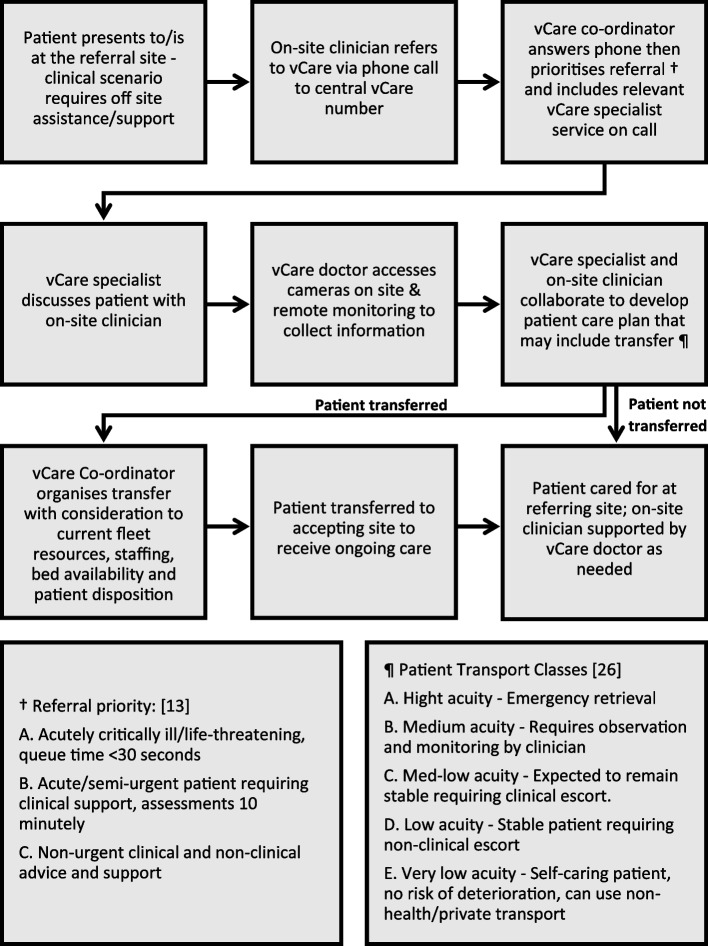
Visual representation of a vCare encounter

vCare is staffed by nursing and medical staff with specialised critical care training (Fellow of Australian College Emergency Medicine or equivalent). Specialists from tertiary referral networked hospitals (MM1) including Newborn and Paediatric Emergency Transport Service (NETS) are also available for consultation. Each referral to vCare is initiated by rural or remote site staff (medical or nursing) or by VRGS doctors and completing a phone call to the vCare office. A vCare nurse coordinator answers the phone and triages the referral based on patient acuity. The referring clinician is connected with either a vCare Emergency Consultant, medical and/or retrieval specialist as needed. The referring site clinician, vCare clinicians and invited specialists (± patient / carer) communicate via phone, telehealth carts, and overhead cameras in resuscitation bays to support timely patient care and inform clinical decision making [13, 14]. Visual representation of the vCare encounter can be found in Fig. 1.

This study extracted data from electronic medical records (eMR) manually by clinical researchers (EM, CC, and KD) from all vCare patient encounters from 1st February to 31st March 2021. The sample size was chosen pragmatically as it was assumed 1500 consecutive encounters would provide sufficient diversity. The two consecutive months were chosen as they were the most recently completed months at the time data collection commenced. Of the 1779 encounters initially included, 102 were excluded due to missing information (patient file unable to be accessed with information provided from vCare) or duplicate encounters being logged. An initial pilot with a sample of 100 encounters resulted in adjustments to include a wider range of variables and focus variables to only include those that could be reliably assessed to improve accuracy.

Variables extracted included remoteness of sites, referral information, health professional involvement, presenting complaint, triage category, camera use, patient characteristics, transfer information, escalation of therapeutic intervention required and secondary transfer requirements. Only aspects of care completed within the WNSWLHD were collected. For inter-hospital transfers external to the WNSWLHD only the destination was logged.

vCare clinical Nurse Coordinators prioritise referrals upon discussing the situation on the phone with the on-site clinician. Referrals in which clinical advice and support is required are categorised into 3 levels of urgency. These levels are Priority A) acutely critically ill / life threatening, queue time < 30 s, Priority B) Acute and semi-urgent requiring clinical advice and support, reassessing patients at 10-min intervals, and Priority C) Non urgent clinical and non-clinical advice and support [13].

Classes of transport were defined in line with New South Wales state policy. There are five transport classes which outline minimum staff and equipment specifications: A) emergency retrieval with a life-threatening clinical scenario, B) Observation and monitoring is required by two specialist escorts, C) Patient expected to remain stable and requires an escort, D) Patient stable with patient transport escort, E) Patient is self-caring with no risk of deterioration and can use a taxi, community transport or private vehicle [13, 26].

The population of each locality serviced by a facility with access to vCare was sourced from the Australian Bureau of Statistics Census data website (2021) [27]. Populations of localities serviced by facilities with a common MM score were aggregated and used as the denominator for calculating vCare encounters per 1000 population.

The principal presenting complaint for each encounter was identified from vCare clinician documentation and classified according to 12 main categories. These categories were developed specifically for this study and separated by bodily system/clinical specialty. They include: gastrointestinal (e.g., abdominal pain, vomiting and diarrhoea), respiratory (e.g., shortness of breath, wheeze and cough), cardiovascular (e.g., chest pain, palpitations, blood pressure-related issues), trauma (e.g., injuries caused by accidents or violence), infection (e.g., fever, abscess, sepsis), unwell child (e.g. unsettled infant or non-specific illness in child), neurology (e.g., seizure, headache, facial or limb weakness, dizziness), obstetrics and gynaecology (e.g., birth or pregnancy related, symptoms related specifically to females), loss of consciousness (regardless of aetiology), musculoskeletal, mental health including drug and alcohol related illness, renal (e.g., dialysis related, electrolyte abnormalities). An additional category of ‘other’ was used for conditions lying outside these categories.

Escalation of care was assessed at three distinct stages; prior to contact with vCare, during referral and post-arrival at the accepting site. The Therapeutic Intervention Scoring System (TISS) tool was used to describe treatment invasiveness, resources and skill set required to complete the care, with level 1 being non-invasive, basic, and monitoring and level 4 being urgent, invasive care such as cardiopulmonary resuscitation. This tool has been previously validated in emergency and intensive care settings for such type of assessment [28–30].

Secondary transfers refer to patients who first undergo an initial transfer to an accepting site coordinated by vCare but are then required to undergo a second transfer to a higher level of care hospital. The second hospital was more appropriately resourced to manage the patient’s underlying illness and provide definitive care. This did not include patients who required a transfer post-discharge or an outpatient procedure at a higher level of care hospital. Data was collected in REDCAP®. Data were analysed in Microsoft Excel© and GraphPad-prism using descriptive statistics focusing on counts, percentages, and cross tabulations. Most percentages in tables were calculated along the vertical axis except where it is noted otherwise. Missing data were excluded.

As a retrospective study, we attempted to limit sampling bias by ensuring that consecutive encounters were assessed. Measurement bias was addressed by using multiple data collectors who collaborated on data collection dilemmas and the use of a non-standardised abstraction tool [31].

## Results

In total, 1678 encounters were included across 38 healthcare facilities supporting a population of over 280,000 people (Table 1). Of these, over 70% had an MM score of 5–7 (rural to remote), with just over half being MM5 (rural). By MM score, the vCare encounters per 1,000 population was highest for MM6 (20.4/1000) and MM7 (17.6/1000) sites and lowest for MM3 (0.2/1000) sites.

**Table 1:** Population, health service facilities and characteristics of vCare encounters by facility remoteness, Western NSW, 1 February to 31 March 2021

	**Modified Monash Model Score**^a^					**Total** **N (%)**
	**3**	**4**	**5**	**6**	**7**	
**Western NSW Health Service**						
Facilities, N (% of row total)	4 (10.0)	4 (10.0)	21 (52.5)	8 (20.0)	3 (7.5)	40 (100.0) 283,060
Population served, N	142,001	62,117	58,501	16,745	3,696	
**vCare encounters**						
N (% of row total)	23 (1.4)	443 (26.5)	800 (47.8)	341 (20.4)	65 (3.9)	1,672 (100.0)
per facility	7.7	110.8	38.1	42.6	32.5	44.0
per 1,000 population	0.2	7.1	13.7	20.4	17.6	5.9
**vCare encounters by sex, % (% of population by sex)**						
Female	47.8 (50.6)	52.6 (50.1)	48.6 (50.0)	48.1 (49.5)	44.6 (50.2)	49.4 (50.3)
Male	52.2 (49.4)	47.4 (49.9)	51.4 (50.0)	51.9 (50.5)	55.4 (49.8)	50.6 (49.7)
**vCare encounters by age, % (% of population by age)**						
Child (< 15 years)	0.0 (20.5)	17.2 (19.4)	10.4 (18.9)	12.3 (19.7)	12.3 (21.6)	12.5 (19.9)
Adult (15 < 65 years)	65.2 (61.7)	48.5 (58.3)	46.4 (56.8)	59.5 (59.9)	64.6 (63.5)	50.6 (59.8)
Older person (65 + years)	34.8 (17.9)	34.3 (22.3)	43.3 (24.4)	28.2 (20.4)	23.1 (15.0)	36.9 (20.3)
**vCare encounters by priority**^b^**, N (%)**						
A (high urgency)	< 5 (16.7)	19 (4.5)	36 (4.6)	17 (5.1)	< 5 (3.2)	77 (4.8)
B (semi-urgent)	8 (44.4)	193 (46.1)	432 (55.3)	160 (47.9)	37 (58.7)	830 (50.1)
C (non-urgent)	7 (38.9)	207 (49.4)	313 (40.1)	157 (47.0)	24 (38.1)	708 (42.7)
**vCare encounters by time of day, N (%)**						
Morning (0700–1430 h)	7 (30.4)	164 (37.1)	256 (32.0)	126 (37.1)	14 (21.5)	567 (34.0)
Afternoon/evening (1430–2300 h)	7 (30.4)	218 (49.3)	433 (54.1)	172 (50.6)	39 (60.0)	869 (52.4)
Night (2300–0700 h)	9 (39.1)	60 (13.6)	111 (13.9)	42 (12.4)	12 (18.5)	234 (14.1)
**vCare encounters by camera assistance, N (%)**						
Yes	< 5 (4.3)	42 (9.5)	186 (23.5)	76 (22.4)	13 (20.3)	318 (19.2)
No	22 (95.7)	398 (90.5)	605 (76.5)	263 (77.6)	51 (79.7)	1,339 (80.8)
**vCare encounters by referring health professional, N (%)**						
Nurse (onsite)	< 5 (14.3)	23 (5.3)	277 (34.9)	76 (22.8)	5 (7.8)	384 (23.3)
Doctor (onsite)	17 (81.0)	414 (94.5)	405 (51.1)	213 (64.0)	56 (87.5)	1,105 (67.0)
VRGS doctor (off-site)^c^	< 5 (4.8)	< 5 (0.2)	111 (14.0)	44 (13.2)	< 5 (4.7)	160 (9.7)
**vCare transfers, N (%)**						
Yes	18 (78.3)	292 (65.9)	558 (69.8)	231 (67.7)	45 (69.2)	1,144 (68.4)
No	5 (21.7)	151 (34.1)	242 (30.3)	110 (32.3)	20 (30.8)	528 (31.6)

*N* number of, *VRGS* Virtual Rural Generalist Service

^a^Measures remoteness and population size on a scale from 1–7 where 1 = major city and 7 = very remote [8)

^b^Referral priority – Incoming Classification Matrix (three urgency levels] [13]

A. Acutely critically ill/life-threatening, queue time < 30 s

B. Acute/semi-urgent patient requiring clinical support, assessments 10 minutely

C. Non-urgent clinical and non-clinical advice and support

^c^VRGS stands for Virtual Rural Generalist Service which is a 24/7 service which utilizes telehealth to support small rural hospitals without medical cover to handle day to day medical care [23]

From the vCare encounters, children (< 15 years old) (12.5%), adults (15–65 years old) (50.6%) and older people (> 65 years old) (36.9%) were evenly split between sexes, and this trend was seen across all hospitals regardless of MM Score (Table 1). These percentages seem to reflect similarly to the general population.

Most incoming referrals (52.4%) occurred in the afternoon/evening (2.30 pm-11 pm), with the most remote sites recording the largest proportion of referrals (60.0%) during this period (Table 1). Most referrals to vCare were requested by on site doctors (67.0%), though there was an increase in referrals by VRGS and nurses in MM5 (34.9% and 14% respectively) and MM6 (22.8% and 13.2%) hospitals.

Incoming vCare referrals were most commonly classed as semi-urgent (51.4%), followed by non-urgent referrals (43.8%) (Table 2). Most incoming referrals were prioritised to the correct urgency level. This was deduced as the priority category of the referral was the same level of urgency as the transfer category. Of critically ill referral (Priority A), 57.4% of these patients requiring transfer to a higher level of care were classed as high-urgency referrals (Transport class A) and only one referral classified as non-urgent but then required urgent transport.

**Table 2:** Characteristics of vCare encounters by referral priority (urgency level), Western NSW, 1 February to 31 March 2021

	**Referral Priority (Urgency level)**^a^			**Total** **N (%)**
	**A (High urgency)**	**B (Semi-urgent)**	**C (Non-urgent)**	
**vCare encounters, N (% of row total)**	78 (4.8)	832 (51.4)	709 (43.8)	1,619 (100.0)
**Time from presentation to referral, N (%)**				
< 1 h	48 (67.6)	327 (41.7)	178 (27.2)	553 (36.6)
1–5 h	18 (25.4)	341 (43.5)	332 (50.8)	691 (45.8)
> 5 h	5 (7.0)	116 (14.8)	144 (22.0)	265 (17.6)
**Camera-assisted referral, N (%)**				
Yes	45 (57.7)	215 (26.1)	58 (8.3)	318 (19.8)
No	33 (42.3)	610 (73.9)	644 (91.7)	1,287 (80.2)
**Transfer priority**^b^**, N (%)**				
A (High)	39 (57.4)	28 (4.4)	1 (0.2)	68 (6.0)
B (Moderate)	29 (42.6)	481 (76.3)	151 (34.2)	661 (58.0)
C (Low-moderate)	0 (0.0)	50 (7.9)	120 (27.2)	170 (14.9)
D (Low)	0 (0.0)	10 (1.6)	38 (8.6)	48 (4.2)
E (Self§)	0 (0.0)	61 (9.7)	131 (29.7)	192 (16.9)
Total transfers (% of encounters)	68 (87.2)	630 (75.7)	441 (62.6)	1,139 (70.5)
Nil transfers (% of encounters)	10 (12.8)	202 (24.3)	264 (37.4)	476 (29.5)
**Presenting condition type, N (%)**^c^				
Cardiac	21 (8.1)	178 (68.5)	61 (23.5)	260 (16.1)
Trauma	< 5 (1.6)	98 (40.2)	142 (58.2)	244 (15.1)
Gastroenteric	< 5 (1.3)	98 (41.0)	138 (57.7)	239 (14.8)
Respiratory	16 (12.3)	81 (62.3)	33 (25.4)	130 (8.0)
Infection	6 (5.0)	52 (43.7)	61 (51.3)	119 (7.4)
Unwell child	< 5 (1.8)	66 (59.5)	43 (38.7)	111 (6.9)
Neurological	5 (4.6)	63 (58.3)	40 (37.0)	108 (6.7)
Obstetric/Gynaecological	0 (0.0)	44 (60.3)	29 (39.7)	73 (4.5)
Syncope/Loss of consciousness	11 (15.9)	38 (55.1)	20 (29.0)	69 (4.3)
Musculoskeletal	< 5 (2.4)	13 (31.7)	27 (65.9)	41 (2.5)
Renal	< 5 (2.9)	17 (48.6)	17 (48.6)	35 (2.2)
Mental Health/Drug & Alcohol	< 5 (11.8)	19 (55.9)	11 (32.4)	34 (2.1)
Other	< 5 (2.6)	65 (41.7)	87 (55.8)	156 (9.6)

^a^Referral priority – Incoming Classification Matrix (three urgency levels] [14]

A. Acutely critically ill/life-threatening, queue time < 30 s

B. Acute/semi-urgent patient requiring clinical support, assessments 10 minutely

C. Non-urgent clinical and non-clinical advice and support

^b^Patient Transport Classes [25]

A. Emergency retrieval for life-threatening clinical scenario (Emergency Ambulance Services only). High acuity

B. Patient requires observation and monitoring by Registered Nurse or Paramedic > Medium acuity

C. Patient expected to remain stable requiring clinical escort. Medium to low acuity

D. Stable patient requiring non-clinical transport escort. Medium to low acuity

E. Self-caring patient, no risk of deterioration, can use non-health/private transport. Low acuity

^c^Percentages by referral priority are calculated using the row total as the denominator, percentages for the Total column use column total as the denominator

The most common presentations generating a vCare referral were trauma (15.1%), cardiac (16.1%), gastroenterological (14.8%) and respiratory (8.0%) in nature (Table 2). The most common reasons for high-urgency referrals included cardiac and respiratory presentations. Mental health and drug and alcohol accounted for a small (2.1%) proportion of referrals, yet of those 25 referrals, 5 required high priority transfer (20%).

Cameras were used 19.8% of the time during vCare encounters, with most (86.5%) occurring at the more remote (MM5-7) sites (Table 1). Most high-urgency referrals (57.7%) utilised overbed cameras to examine and view patients, additionally, 57.4% of high-urgency referrals also required high transfer priority (Table 2).

### During patient transfer

Of the 1678 vCare encounters assessed, 1144 (68.4%) required transfer (Table 1). Of M4-7 encounters, 66–70% were transferred compared to 78% of M3 encounters. The most frequent transfers were those of moderate priority (59.8%) (Table 2). The proportion of transfers increased as referral urgency increased with 87.2% of high-urgency referrals being transferred compared to 62.6% of non-urgent referrals (Table 2). However, while most high-urgency referrals (57.4%) had high transfer priority, a large proportion (42.6%) had only moderate transfer priority.

### Post-patient transfer

The most common accepting sites were the three major regional hospitals located in MM3 locations—Dubbo (58.2%), followed by Orange (28.7%) and Bathurst (7.8%). The vast majority (95.1%) of transfers were accepted by these facilities within the study region (Table 3). Only 2.5% of all patients and 7.8% of patients with life-threatening conditions (e.g., severe trauma that required high transfer priority) were transferred outside of the local health district (Table 3). Transfers from Procedural hospitals accounted for 2.5% of transfers. Metropolitan hospitals (MM1) accounted for 2.5% of accepting sites for the initial transfer but accounted for 54.1% of secondary transfers (Table 1).

**Table 3:** Characteristics of vCare encounters at accepting sites by transfer priority, Western NSW, 1 February to 31 March 2021

	**TRANSPORT CLASSIFICATION (Urgency Level)**^a^					**TOTAL** **N (%)**
	**A (High)**	**B (Mod)**	**C (Low-mod)**	**D (Low)**	**E (Self)**	
**Transferred vCare encounters, N (% of row total)**						
	64 (5.9)	646 (59.8)	161 (14.9)	37 (3.4)	172 (15.9)	1,080 (100.0)
**Transfer accepting site, N (%)**						
MM3 facility in study region	59 (92.2)	644 (95.7)	157 (94.0)	40 (95.2)	179 (94.7)	1,079 (95.1)
Other within study region^b^	0 (0.0)	10 (1.5)	7 (4.2)	< 5 (4.8)	9 (4.8)	28 (2.5)
Other outside study region^c^	5 (7.8)	19 (2.8)	< 5 (1.8)	0 (0.0)	< 5 (0.5)	28 (2.5)
**Specialty team at accepting site, N (%)**						
Physician care^d^	23 (41.0)	271 (42.1)	43 (26.4)	5 (11.9)	14 (8.1)	356 (33.1)
Emergency Department^f^	< 5 (5.4)	124 (19.3)	43 (26.4)	16 (38.1)	57 (33.1)	243 (22.6)
Surgical care^e^	< 5 (1.8)	113 (17.5)	61 (37.4)	14 (33.3)	62 (36.0)	251 (23.3)
Paediatrics	< 5 (1.8)	50 (7.8)	5 (3.1)	6 (14.3)	29 (16.9)	91 (8.4)
Intensive Care/High Dependency	22 (39.3)	41 (6.4)	5 (3.1)	0 (0.0)	< 5 (1.2)	70 (6.5)
Obstetrics/Gynaecology	< 5 (5,4)	37 (5.7)	< 5 (1.8)	< 5 (2.4)	6 (3.5)	50 (4.6)
Psychiatry or Drugs & Alcohol	< 5 (5.4)	8 (1.2)	< 5 (1.8)	0 (0.0)	< 5 (1.2)	16 (1.5)
**Imaging at accepting site, N (%)**						
Nil used	9 (15.8)	138 (21.5)	23 (14.0)	15 (35.7)	67 (39.4)	252 (23.4)
CT, MRI &/or Echocardiography^g^	25 (43.9)	237 (36.9)	87 (53.0)	12 (28.6)	39 (22.9)	400 (37.2)
Ultrasound or X-ray	10 (17.5)	165 (25.7)	44 (26.8)	14 (33.3)	59 (34.7)	292 (27.2)
Imaging Angiography	< 5 (3.5)	59 (9.2)	9 (5.5)	< 5 (2.4)	5 (2.9)	76 (7.1)
Coronary Angiography	11 (19.3)	43 (6.7)	< 5 (0.6)	0 (0.0)	0 (0.0)	55 (5.1)
**Length of stay at accepting site, N (%)**						
< 24 h	9 (15.8)	196 (30.6)	48 (29.4)	18 (42.9)	90 (53.6)	361 (33.7)
24 h – 7 days	29 (50.9)	336 (52.5)	86 (52.8)	19 (45.2)	68 (40.5)	538 (50.3)
> 7 days	19 (33.3)	108 (16.9)	29 (17.8)	5 (11.9)	10 (6.0)	171 (16.0)
**Secondary transfer from accepting site, N (% of ‘Transferred vCare encounters’)**						
Nil secondary transfer	49 (89.1)	559 (86.9)	141 (85.5)	42 (95.5)	162 (94.2)	953 (88.3)
Metropolitan facility outside study region	5 (9.1)	53 (8.2)	9 (5.5)	0 (0)	6 (3.5)	73 (6.8)
Higher care facility within study region	1 (1.8)	31 (4.8)	15 (9.1)	2 (4.5)	4 (2.3)	53 (4.9)

*Mod* Moderate, *MM* Modified Monash Model, *Metro* Metropolitan, *CT* Computerized tomography, *MRI* Magnetic resonance imaging

^a^Patient Transport Classes [26]

A. Emergency retrieval for life-threatening clinical scenario (Emergency Ambulance Services only). High acuity

B. Patient requires observation and monitoring by Registered Nurse or Paramedic > Medium acuity

C. Patient expected to remain stable requiring clinical escort. Medium to low acuity

D. Stable patient requiring non-clinical transport escort. Medium to low acuity

E. Self-caring patient, no risk of deterioration, can use non-health/private transport. Low acuity

^b^More than 80% of 'Other within study region' transfers were accepted by MM4 (procedural) facilities

^c^Nearly 90% of 'Other outside study region' transfers were accepted by metropolitan facilities

^d^ Includes cardiology, respiratory, oncology, renal, neurology and general medicine

^e^Includes general surgery, urology and ear, nose, and throat (ENT) surgery

^f^Only required assessment and/or treatment in the Emergency Department and was not admitted by a specialty team

^g^CT, MRI &/or echocardiography total = 410; *N* = 391 at MM3 sites and *N* = 14 at MM4 sites

Most patients (50.3%) remained at the accepting site between 24 h and 7 days. In general, as urgency increased, the proportion of encounters with LOS less than 24 h decreased while that for LOS greater than 7 days increased (Table 3). The need for surgical care (23.3% of transfers) and physician care (33.1% of transfers) were the most common reasons for admission at accepting locations. However, for high priority transfers, 39.3% were treated in Intensive Care Units. Almost a quarter (22.6%) of transfers only received care in the emergency department. The main imaging modalities utilised were magnetic resonance imaging (MRI) and computed tomography (CT) scans (37.2%) followed by ultrasound (US) and X-ray (27.2%). More invasive imaging (e.g., angiography) was more likely to be used following high priority transfer.

**Fig. 2:**
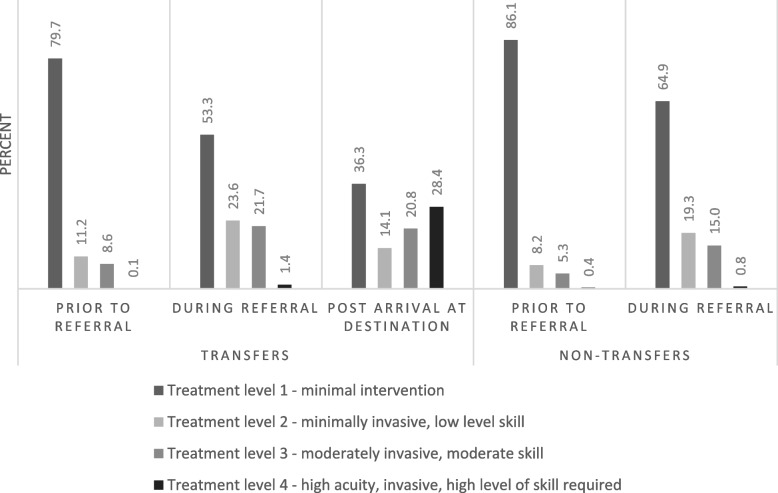
Treatment level 1 provided during vCare encounters by transfer and referral status three timepoints as characterised by the TISS-76 score which categories interventions based on resources, invasiveness, and skill [28–30], Western NSW, February–March 2021

With each successive time point—prior to referral, during referral and post-arrival at destination – the level of therapeutic intervention escalated. Of patients who were not transferred, prior to the initiation of the referral to vCare 86.1% of patients received level 1 care (minimally invasive, observation level), which decreased to 64.9% during referral (Fig. 2). Accordingly, the rates of level 2–4 care (more invasive and urgent interventions) all rose after initiation of referral to vCare – level 2 care by 11.1%, level 3 care by 9.7% and level 4 care by 0.4%. Of patients who were transferred, level 1 care decreased at each of the three time points recorded – dropping from 79.7% prior to referral to 53.3% during referral and then 36.3% post-arrival. Level 2 care rose from time point 1 to time point 2 by 12.3% but dropped to 14.1% post-arrival. Level 3 and 4 care both rose at each time point to reach 20.8% and 28.4% post-arrival. Out of all vCare encounters, < 5% did not have medical support during the referral. vCare doctors provided support most of the time (67.8%), except in MM3 hospital referrals (4.8%).

### Survival

Almost all (98.6%) of patients survived the episode of care during which they had an encounter with vCare. No deaths occurred while waiting or during transfer. Seven deaths occurred at the referring site whilst vCare was supporting staff on the ground virtually to treat the patient and 17 deaths occurred post-transfer to the accepting site, with two of those patients dying within 24 h of arrival (Table 4).

**Table 4:** vCare patient survival status before, during and after transfer, Western NSW, February–March 2021

Survival status	Patients, N (%)
Survived	1,643 (98.6)
Died	24 (1.4)
At referring site	7 (0.4)
Awaiting transfer	0 (0.0)
During transfer	0 (0.0)
≤ 24 h at accepting site	< 5 (0.1)
> 24 h at accepting site	15 (0.9)

## Discussion

Our retrospective observational study described the vCare patient journey from call initiation at the referring site to definitive care at the accepting site. Remote locations (MM4-6) used vCare at a higher rate than regional facilities. The type of clinician referring to vCare was more likely to be a nurse or VRGS doctor in increasingly remote sites, compared to doctor-led referrals in regional sites. This is likely a reflection of multiple compounding inequities in remote settings including the universal challenges of rural/remote workforce, and that facilities in remote settings are not role delineated to manage severely unwell patients for extended periods [4–6, 12]. vCare provided care for patients of all ages and sexes (Table 1) in a similar way to in-person presentations of Australian Emergency Departments [32], therefore rising to the challenge of providing rural and remote communities with access to appropriate healthcare.

Cameras were installed in resuscitation bays in 2008–2010 to provide an adjunct to the telephone conferencing technology, allowing visual communication to remote sites and clinicians [13, 14]. Camera-utilisation as an adjunct across all facility-types was unexpectedly low. Camera use was highest in remote sites and increased with referral urgency / acuity as is expected given vCare camera activation criteria when managing a critically unwell patient [14]. Previous studies have shown that optimal communication is vital for success of telehealth in critical care situations and so we feel this would limit the effectiveness of the vCare service [16, 19, 33]. Further investigation is required to understand camera-assistance variances and whether rural-remote patients and clinicians could be better served by utilising other technologies. This is especially important as telehealth is more heavily relied upon, especially in rural areas, to address resource and workforce limitations [7].

A clinically and culturally significant component of vCare is to, where possible, allow patients to remain close to home or on country (refers to the cultural importance for Aboriginal peoples to remain on the land of their ancestors due to their deep spiritual connection to the environment), including for end-of-life situations [34]. Of the vCare encounters reviewed, one third did not require transfer to another facility, supporting patients in receiving care close to home. For these patients, it should be noted that the level of intervention increased suggesting vCare was able to support an appropriate escalation of care post-referral (Fig. 2). However, it is worth noting, the suitability of the 68% of patients requiring transfer was not included in this study and an opportunity to look at for future studies.

Despite the urgency status, a large proportion of high-urgency referrals were not transferred using high priority Class A transport – emergency aerial or road resource for transfer, such as NSW Ambulance [26]. Possible reasons include access on demand to emergency and non-emergency transport resources (such as vehicles, workforce, and supplies) and vast distances required to be travelled. Alternatively, this could be due to the escalation of care after initiation of the vCare referral leading to patient stabilisation and allowing downgrading of transport resource requirements. vCare demonstrated efficacy through high survival rates. While high survivability is expected with healthcare services, this is important because patients who are referred to vCare are often critically unwell. Of the patients who did not survive, there were none who died whilst waiting for transport at the remote site or during transfer.

The most common vCare transfers were due to presentations for trauma, cardiac and gastrological conditions with most care being provided by medical, surgical, and emergency specialties at the accepting site. vCare transferred patients to major regional hospitals (MM3) 95% of the time, reflecting appropriate utilisation of local resources within WNSWLHD and avoiding unnecessary transfer to metropolitan facilities out of area. This correlates well with the NSW Rural Health Plan (2014) to “improve access to health services as close to home as possible” [35], with a substantial proportion of transferred patients received CT, MRI and/or echocardiography, predominantly at regional (MM3) sites. WNSWLHD also has multiple procedural facilities (MM4) with access to a limited range of workforce, imaging, and pathology resources. vCare only utilised these sites in 2.5% of transfer, which may suggest an underutilisation of these facilities. This study did not review what proportion of patients may have been suitable to receive care at these procedural sites, however this is an opportunity for future research.

Of the patients who received care at procedural sites, only a small proportion (< 5%) required imaging. Imaging is an important part of the diagnostic process and essential for managing patient care and safety [7, 11, 36]. Local access to imaging reduces demand for larger facilities and transport systems. The Australian Government has previously acknowledged the lack of diagnostic imaging available in rural areas [11]. The low utilisation of imaging at these sites again may point to an underutilisation of the procedural facilities within the WNSWLHD referral network however given that these sites have no onsite emergency surgery capacity, there may be appropriate clinical considerations as to why these sites only received a low proportion of referrals from smaller rural facilities.

Secondary transfers to facilities offering a higher level of care occurred in under 10% of encounters reviewed. A delay in definitive treatment and an improper transfer can hinder patient safety and cause unnecessary expenditure by the healthcare system [37, 38]. Yet, we must also accept that it cannot always be possible to transfer patients to definitive care 100% of the time. Patient condition may deteriorate and the need for higher level intervention and treatment may escalate as further investigations reveal new information [39]. Therefore, a small proportion of secondary transfers is expected but taking steps to minimise this is key in using resources prudently [35–37]. Further studies would be required to determine whether secondary transfers could be further lowered prior to initial transfer.

While transfers from a rural hospital to a regional/metropolitan hospital have been shown to improve patient outcomes in a variety of life-threatening scenarios [40], multiple studies have demonstrated that unnecessary transfers can be averted with the use of telehealth [15, 41, 42] as well as improve the quality of care received by patients on site [19, 43]. Each transfer is costly with time, personnel, resources and financially, estimated between $2000-$6000 for the healthcare system as well as disruption for patients and families [41–46]. vCare supported local care through facilitating timely advice and supporting clinicians to manage patients on site with available resources in 31.6% of cases.

### Limitations

As a retrospective analysis, the availability and reliability of the data collected was limited to the data captured on patients’ electronic medical records. There was no control group to compare this service to which limited the assessment that could be completed. Notwithstanding, the results describe a service which provides varied and vital services to a poorly resourced community in regional and rural Australia. We created categories for presenting complaint based on the specialty teams the patient was being referred to. Data were collected at call initiation and therefore prior to formal diagnoses where ICD-10 codes could be applied. While a more universally recognised category may have enhanced generalisation, the temporal nature of the presenting complaint is more clinically relevant.

The period assessed in this study was chosen due to convenience, yet it is important to acknowledge the bias this could have introduced to our data. Given that two contiguous months were chosen at the end of summer, one must consider the possibility of seasonality bias [47]. The chosen months also occurred during the COVID-19 pandemic and this likely introduced additional bias, such as overrepresentation of respiratory illnesses and possible prejudice for transfer decisions. The area studied was not in lockdown during the study period and the number of ED presentations across the state remained stable compared to the previous year however we recognise this carries a risk of bias [48, 49].

## Conclusion

vCare was used by staff in rural and remote facilities to support clinical decision-making for patients in a critical condition. Care was provided at the referring site for a third of the patients and escalation of care and transport of patients occurred in two thirds of referrals. Issues identified included low utilisation of camera equipment, heavy reliance on regional sites and high rates of secondary transfer. vCare has delivered a regional solution to providing emergency advice and retrieval in a large rural and remote area experiencing workforce shortages and limited resources. Through description of vCare and identification of its nuances we hope to inspire other geographically dispersed countries to consider similar services to meet healthcare needs of their communities.

### Further study

More detailed analysis on the effectiveness of the service is still warranted, including identifying factors leading to error and poor outcomes as well as recognising optimum use of resources. Further study is also needed in multiple areas of this developing service, such as diagnostic and treatment accuracy, precise timing of transfers, incongruent transfers, causes of delays and qualitative analysis of clinicians and patients’ experiences utilising the service.

## Data Availability

Datasets generated and analyzed during this study are not publicly available in line with ethics approval from Greater Western Health Research Ethics Committee (GWHREC). These data are not publicly available because of the rural and remote setting of the study, where several small sites contributed small numbers of patients to the study. The consequence of this is that de-identified data presented in disaggregated form may lead to identification of individuals in small sites. Further access for secondary use of data would require specific ethical approval from the GWHREC and the Western NSW Local Health District. Requests to access data can be made to Kimberley Dean.

## Abbreviations

Accepting site: Healthcare facility receiving transfer of patient from their original location
Clinicians: Healthcare professionals including medical practitioners, nurse practitioners and nurses (both enrolled and registered)
CT: Computed tomography
ED: Emergency department
MM: Modified Monash Model
MRI: Magnetic resonance imaging
NETS: Newborn and paediatric emergency transport service
NSW: New South Wales
Procedural site: A type of hospital with medical practitioner and nursing coverage at all hours as well as basic imaging and diagnostic pathology. Therefore, can complete basic procedures.
Referral: Communication between vCare and the clinicians at the initial presenting site of the affected patient
Referring site: The original healthcare facility at which the patient presented who contacted vCare for assistance
Retrieval medicine: Assessing, stabilising, and transporting patients with severe injury or critical illness [24]
Rural Generalist: A medical practitioner who is a general practitioner with additional skills in emergency medicine, obstetrics and gynaecology, paediatrics, or other specialties
Secondary transfer: Patients who require a second transfer to a higher acuity hospital after their initial transfer to an accepting site coordinated by vCare
Telehealth: Provision of medical services through secure telephone or video-conferencing technology [25]
TISS: Therapeutic Intervention Scoring System
Transfer: The relocation of a patient from the referring site to the accepting site [12]
Transport: The physical act of carrying a person or good from one place to another by means of a vehicle, aircraft, or ship
US: Ultrasound
vCare: Virtual Coordination, access, referral, escalation service
VRGS: Virtual Rural Generalist Service
WNSWLHD: Western NSW Local Health District
